# Interindividual heterogeneity affects the outcome of human cardiac tissue decellularization

**DOI:** 10.1038/s41598-021-00226-5

**Published:** 2021-10-21

**Authors:** Miguel F. Tenreiro, Henrique V. Almeida, Tomás Calmeiro, Elvira Fortunato, Lino Ferreira, Paula M. Alves, Margarida Serra

**Affiliations:** 1 iBET, Instituto de Biologia Experimental e Tecnológica, Apartado 12, 2781-901 Oeiras, Portugal; 2 Instituto de Tecnologia Química e Biológica António Xavier, Universidade Nova de Lisboa, Avenida da República, 2780-157 Oeiras, Portugal; 3 CENIMAT|i3N, Departamento de Ciência dos Materiais, Faculdade de Ciências e Tecnologia, Universidade NOVA de Lisboa, Campus de Caparica, 2829-516 Caparica, Portugal; 4 CNC, Centro de Neurociências e Biologia Celular, Universidade de Coimbra, 3004-517 Coimbra, Portugal; 5 Faculdade de Medicina, Universidade de Coimbra, Rua Larga, 3004-504 Coimbra, Portugal

**Keywords:** Biomaterials, Tissue engineering, Pluripotent stem cells

## Abstract

The extracellular matrix (ECM) of engineered human cardiac tissues corresponds to simplistic biomaterials that allow tissue assembly, or animal derived off-the-shelf non-cardiac specific matrices. Decellularized ECM from human cardiac tissue could provide a means to improve the mimicry of engineered human cardiac tissues. Decellularization of cardiac tissue samples using immersion-based methods can produce acceptable cardiac ECM scaffolds; however, these protocols are mostly described for animal tissue preparations. We have tested four methods to decellularize human cardiac tissue and evaluated their efficiency in terms of cell removal and preservation of key ECM components, such as collagens and sulfated glycosaminoglycans. Extended exposure to decellularization agents, namely sodium dodecyl sulfate and Triton-X-100, was needed to significantly remove DNA content by approximately 93% in all human donors. However, the biochemical composition of decellularized tissue is affected, and the preservation of ECM architecture is donor dependent. Our results indicate that standardization of decellularization protocols for human tissue is likely unfeasible, and a compromise between cell removal and ECM preservation must be established in accordance with the scaffold’s intended application. Notwithstanding, decellularized human cardiac ECM supported human induced pluripotent-derived cardiomyocyte (hiPSC-CM) attachment and retention for up to 2 weeks of culture, and promoted cell alignment and contraction, providing evidence it could be a valuable tool for cardiac tissue engineering.

## Introduction

Engineering biomimetic cardiac tissue is still challenging, and even the most promising approaches reported to date fail to integrate all the key components present in the cardiac niche, namely a sufficiently complex extracellular matrix (ECM). These engineered tissues often rely on biomaterials for tissue assembly^1^, but such biomaterials are either simplistic compared to the ECM (e.g., single-protein matrices with collagen or fibronectin), have little or no presence in vivo (e.g., fibrin or synthetic polymers), bear batch-to-batch variability (e.g., Matrigel®), and are often from animal origin (e.g., rat-tail collagen, porcine gelatin or Matrigel®). Even though bioartificial cardiac tissues are promising platforms to expedite drug development, their use as reliable disease models merits improvements as the cardiac ECM plays a critical role in several cardiac pathologies, such as myocardium infarction and progression into heart failure^2^. Alternative sources to cardiac ECM with clearance for medical applications are mostly from animal origin, such as porcine small intestine submucosa (SIS) and porcine urinary bladder matrix^3^. Lack of cardiac specificity of alternative ECM sources has shown to influence embryonic stem cell differentiation towards cardiomyocytes^4^. This may be due to a differential ECM biochemical profile and architecture between tissues/organs, that ultimately affects stem cell fate and optimal cell function. Naturally derived animal cardiac ECM can improve these shortcomings and has shown to contribute to superior cardiomyocyte performance^4,5^. Nonetheless, human-specific features of cardiac ECM might be lacking in products from animal origin, and so a human derived biomaterial could, in principle, more closely resemble the cardiac microenvironment, besides circumventing issues related with xenogenecity.

Decellularization is the standard methodology to obtain an ECM scaffold from tissues and organs, in which ideally all the cellular components and genetic material are removed, leaving a preserved natural scaffolding biomaterial with a native architecture suitable for tissue/organ bioengineering. Most studies rely on whole-heart decellularization to obtain cardiac ECM, that uses perfusion through the organ vasculature to infuse in a stepwise manner the decellularization solutions (typically chemical or enzymatic)^6–8^. Initial studies showed that anterograde coronary perfusion with 1% of the ionic detergent sodium dodecyl sulfate (SDS) for 12 h was sufficient to fully decellularize a rat heart^6^; however, if detergent time is kept constant (12 h), decellularizing a porcine heart requires 4% SDS^9^, otherwise longer periods of SDS exposure (at 1%) are required, as it is the case of the human heart (192 h)^8^. Therefore, inter-species differences, including recognizable sizing variations, are likely to compromise direct translation of successful protocols optimized in animals for human use. Furthermore, perfusion-based decellularization protocols are suitable to decellularize *postmortem* hearts unfit for transplantation but difficult to use accurately to decellularize tissue samples from cardiac biopsies, that are also more easily obtained from human donors undertaking interventional surgery. These tissue samples can be decellularized with the same decellularization agents using agitation instead, but established protocols in small or large animals may prove difficult to adapt to human tissue without additional modifications^10,11^. Besides customization of decellularization protocols for human usage, the heterogeneity between donors might prevent their standardization. In fact, decellularizing human liver discs with a protocol optimized for porcine liver decellularization was only successful for one donor, with the other four showing different levels of remaining cells and matrix^12^.

In this work, we evaluated how effective these previous protocols are to decellularize human cardiac tissue in three unrelated human donor samples and assessed the suitability of the decellularized scaffold to support the culture of cardiomyocytes derived from human induced pluripotent stem cells for application in cardiac tissue bioengineering.

## Materials and methods

### Decellularization of human cardiac tissue

Heart tissues were harvested from donor human hearts that were not suitable for transplantation at the Coimbra Hospital and University Centre (CHUC, Coimbra, Portugal), after informed consent was obtained from all patients and in accordance with the regulations of tissue bank and the approval of the Ethics Committee of Medical School of the University of Coimbra (CE-022/2017). All methods in this study involving human heart tissue were carried out in accordance with relevant guidelines and regulations. Donor data and tissue experimental allocation are outlined in Table 1.

**Table 1:** Cardiac tissue donor data. *LV* left ventricle, *hiPSC-CM* human induced pluripotent cardiomyocytes.

Characteristics	Donor 1	Donor 2	Donor 3	Donor 4
Age	69	67	67	61
Sex	Male	Female	Female	Male
Cause of death	Intracerebral hemorrhage	Not disclosed	Not disclosed	Not disclosed
Reason for exclusion from transplantation	Not disclosed	Not disclosed	Not disclosed	Not disclosed
Tissue source	Myocardium, LV; No pathology associated	Myocardium Apex, LV; No pathology associated	Myocardium, LV; No pathology associated	Myocardium, LV; No pathology associated
Experimental allocation	Evaluation of decellularization protocols	Evaluation of decellularization protocols	Evaluation of decellularization protocols	Decellularization Method D; Recellularization with hiPSC-CM

Soon after harvest, tissue samples were stored at − 80 °C and kept there until usage. Myocardial tissue samples were thawed and cut into pieces of about 1 mm in thickness, as previously described^10,11^. The following decellularization methods were modified from already published protocols to assess their applicability for human tissue. For each method tissue pieces were decellularized with the following succession of solutions: Method A) 10 mM Tris (Calbiochem), 5 mM ethylenediamine tetraacetic acid (EDTA, Sigma-Aldrich), pH 7 for 2 h (lysis buffer); 0.5% wt/vol SDS (Sigma-Aldrich) in Dulbecco's phosphate-buffered saline (DPBS, Thermo Fisher Scientific) for 6 h; DPBS overnight; fetal bovine serum (FBS) for 3 h at 37 °C (serum DNAase activity can aid the removal of nucleic acids before rinsing^13^); DPBS for 24 h (modified from^14^); Method B) 1% wt/vol SDS in DPBS for 48 h; 1% vol/vol Triton-X-100 (Sigma-Aldrich) in DPBS for 30 min; DPBS for 24 h (modified from^10^); Method C) 10 mM Tris, 5 mM EDTA, pH 7 for 24 h (lysis buffer); 1% wt/vol SDS in DPBS for 48 h; 1% vol/vol Triton-X-100 in DPBS for 30 min; DPBS for 24 h; Method D) 1% wt/vol SDS in DPBS for 192 h; 1% vol/vol Triton-X-100 in DPBS for 24 h; DPBS for 24 h (modified from^8^). Each step in all methods was performed at room temperature (RT, 20–25 °C), if not stated otherwise, under constant orbital agitation to aid reagent diffusion into the tissue. Gross tissue morphology was imaged in a stereo microscope (DMIRB, Leica).

### Histology and Nuclei fluorescent staining

Tissue pieces were fixed with 4% wt/vol buffered paraformaldehyde overnight, paraffin embedded and sectioned (3 μm thickness) on a rotary microtome (RM 2135, Leica). After deparaffinization, samples were stained with Hematoxylin & Eosin (H&E) (Sigma-Aldrich), Masson Trichrome with Aniline Blue (IHC World), Picro Sirius Red (Polysciences) and Alcian Blue (Polysciences), according to the manufacturer’s instructions. Histological images were digitalized in a NanoZoomer SQ whole slide scanner (Hamamatsu Photonics) and viewed with NDP.view open-source software (NDP.view v.2.7.43, Hamamatsu Photonics). Cell alignment was measured with the FibrilTool Fiji plugin^15^, and the mean angular orientation for each cell-containing ROI was computed across several histological micrographs. Density histograms were generated in R (v.4.0.3) using the ggplot2 package (v.3.3.3) with a bin of 2°. For  nuclei staining microscopy analysis, deparaffinized samples were mounted in ProLong™ Gold antifade reagent containing 4′,6′-diamino-2-fenil-indol (DAPI) (Life Technologies). Samples were viewed in a fluorescence microscope (DMI600, Leica) and DAPI-stained nuclei were quantified in Fiji^15^. The number of nuclei counts were normalized to ROI area.

### Biochemical analysis of cardiac extracellular matrix

Biochemical characterization of cardiac ECM was carried out as previously described^16^. Decellularized samples were digested in 500 μL of papain solution (125 μg mL^−1^ papain in 0.1 M sodium acetate, 5 mM cysteine-HCl, 0.05 M EDTA, pH 6.0, all from Sigma-Aldrich) for 20 h at 60 °C under agitation. Native tissue samples with comparable wet weight were also digested to serve as controls. The dsDNA content was determined using the Quanti-iTTM PicoGreen™ assay (Thermo Fisher Scientific), according to the manufacturer’s instructions. The fluorescent intensity was measured in the microplate reader Infinite®200 PRO NanoQuant (TECAN). The sulfate glycosaminoglycan (s-GAG) content was quantified using the 1,9-dimethylmethylene blue assay (Glycosaminoglycan Assay Blyscan™, Biocolor) and collagen content (soluble and insoluble) was determined via the hydroxyproline assay (Sircol™ Collagen Assay, Biocolor), according to the manufacturer’s instructions. Sample absorbance was measured in the microplate reader Infinite®200 PRO NanoQuant at the corresponding ECM component wavelength. Data was expressed as the measured component mass normalized to tissue wet weight.

### Scanning electron microscopy

Tissue samples were fixed with 2.5% vol/vol buffered glutaraldehyde (Sigma-Aldrich), for 2 h at room temperature. Fixed tissue was washed in DPBS (three times: 5–10 min each) and dehydrated with a series of ethanol solutions, starting with 50% vol/vol and progressing through 70%, 80%, 95% and 100% absolute ethanol. Samples were dried in adhesive carbon tabs (12 mm, Agar Scientific), sputter-coated with a 15 nm Au/Pd film and visualized in a Hitachi TM3030Plus tabletop scanning electron microscope operated in BSE mode at an accelerating voltage of 15 keV. The alignment of ECM fibers was measured with the FibrilTool Fiji plugin. In brief, the mean fiber orientation direction and anisotropy index (0—random alignment/isotropic array, 1—perfect alignment/purely anisotropic array) were computed for each ROI and across several photomicrographs. Density histograms were generated in R (v.4.0.3) using the ggplot2 package (v.3.3.3) with a bin of 15°.

### hiPSC culture and differentiation towards cardiomyocytes

hiPSC line DF19-9-11 T.H (WiCell, Madison, WI) was used in this study. hiPSC were expanded on Matrigel® (Corning) coated plates in mTeSR1 medium (STEMCELL Technologies) at 37 °C, in a humidified atmosphere of 5% CO_2_. The culture medium was exchanged daily and cells were routinely passaged when reaching 80% confluence using Versene (Thermo Fisher Scientific). Differentiation of hiPSC into cardiomyocytes (hiPSC-CM) was induced when cell confluence reached 90%, as previously reported^17,18^. Briefly, at day 0 of differentiation, expansion medium was replaced by RPMI medium (Thermo Fisher Scientific) supplemented with B27 without insulin (RPMI/B27 minus ins., Thermo Fisher Scientific), 80 ng/mL activin A (PeproTec), and 50 mg/mL ascorbic acid (Sigma–Aldrich). After 24 h, spent medium was changed for fresh RPMI/B27 minus ins. supplemented with 5 µM IWR-1 (Sigma–Aldrich) and 50 µg/mL ascorbic acid (Sigma–Aldrich). At day 3 (72 h after induction), spent medium was exchanged for fresh RPMI/B27 minus ins. supplemented with 5 µM IWR-1. Spontaneous beating generally started by day 7 of differentiation.

### Recellularization of decellularized ECM with hiPSC-CM

Decellularized cardiac tissue pieces were immobilized on Poly-l-Ornithine (Sigma–Aldrich) coated coverslips placed on 24-well plates. Briefly, 500 µL of a 16% vol/vol Poly-l-Ornithine solution in DPBS−/− (Thermo Fisher Scientific) was added on top of each coverslip for 3 h and afterwards each well was washed twice with DPBS−/−. Coated plates were left at 4 °C overnight. Decellularized tissue pieces were placed on top of each coverslip for immobilization and washed thrice with 70% vol/vol ethanol for 15 min, followed by UV sterilization in aseptic conditions for 20 min.

Contracting hiPSC-CM monolayers were dissociated by incubation with TrypLE™ Select (Thermo Fisher Scientific) for 5 min at 37 °C, added to culture medium (RPMI/B27 minus ins.), centrifuged at 220×*g* for 5 min, and then used to recellularize tissue scaffolds decellularized through method D. hiPSC-CM (1.25 × 10^6^ cells) were seeded onto each scaffold in a suspension volume of 40 µL under static conditions. After 30 min the tissue was immersed in RPMI medium supplemented with B27 with insulin (RPMI/B27 plus ins., Thermo Fisher Scientific). Medium was changed every 3 days for a total of 14 days of culture.

### Immunofluorescence of recellularized cardiac tissue

Recellularized cardiac tissue was collected from culture and fixed in 4% wt/vol paraformaldehyde with 4% wt/vol sucrose in DPBS for 20 min at RT. Samples were dehydrated in 30% wt/vol sucrose overnight at 4 °C, embedded in Tissue-Tek® O.C.T. (Sakura) and frozen at − 80 °C until use. The blocks of O.C.T. were then sliced with a thickness of 10 μm in a cryomicrotome (Cryostat CM 3050, Leica). Cryosections were allowed to dry at RT for 1 h, permeabilized with 0.1% wt/vol Triton X-100 solution in DPBS and blocked with 0.2% wt/vol Fish Skin Gelatin in DPBS solution (hereafter designated as blocking buffer solution) for 30 min at RT. Primary antibodies (cardiac troponin T, Thermo Fisher Scientific, 1:200) were incubated overnight at 4 °C and secondary antibodies (goat anti–mouse AlexaFluor 488, Thermo Fisher Scientific, 1:500) were incubated for 1 h at RT, both diluted in blocking buffer solution. Samples were mounted with ProLong™ Gold antifade reagent containing DAPI and visualized using a confocal microscope (SP5 Live, Leica).

### Statistics

Data are presented as mean ± standard deviation (SD). Raw data were tested for normality of distribution using the Shapiro–Wilk normality test, and statistical analyses were performed using unpaired t-tests (two-tailed), Kruskal–Wallis tests with Dunnett’s multiple-comparison test or one-way ANOVA tests with Tukey’s multiple comparison test. GraphPad Prism version 7.0a was used for statistical analyses.

## Results

### Differences in human cardiac tissue decellularization outcome across protocol and donor

**Figure 1:**
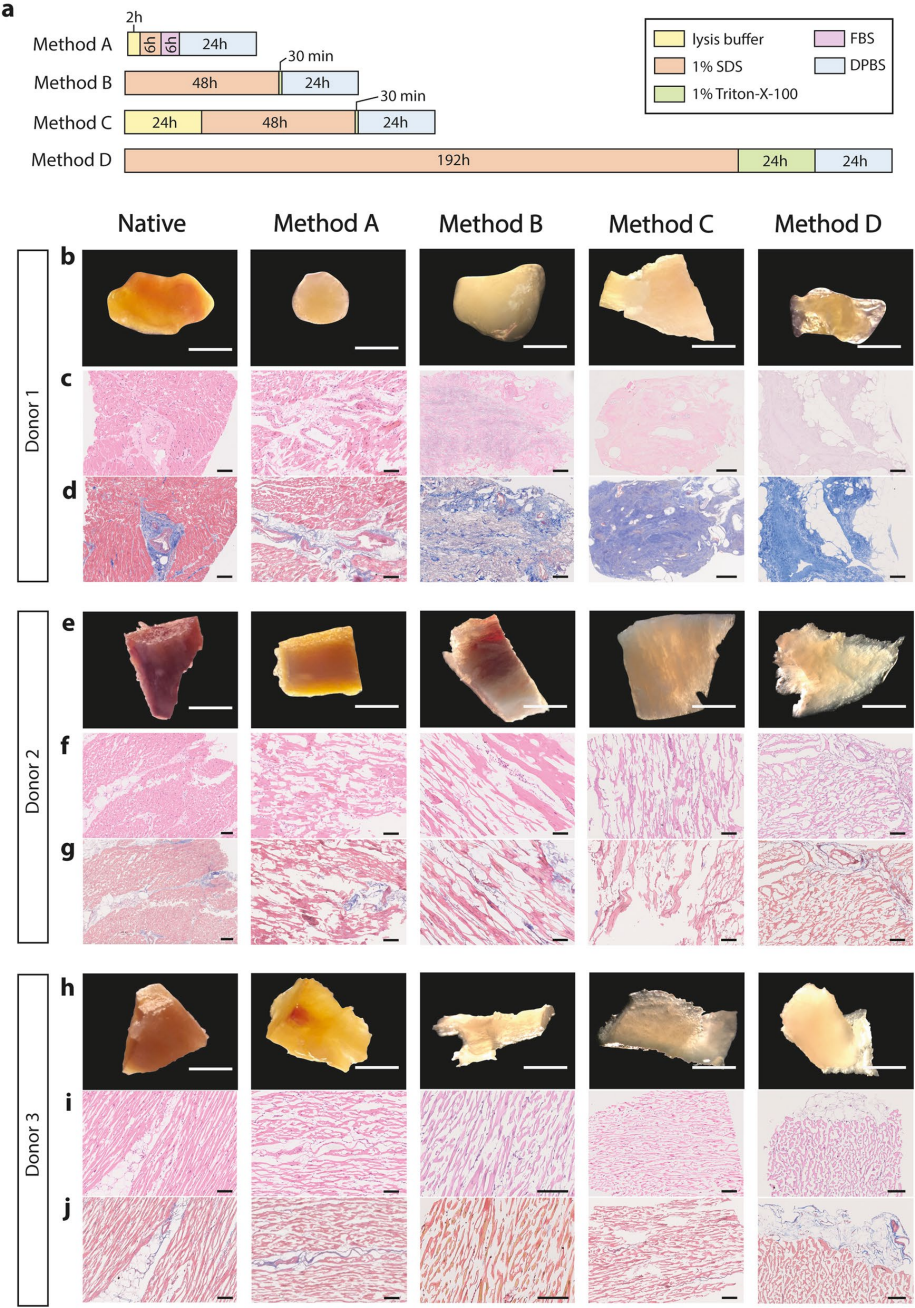
Histological assessment of the different chemical-based decellularization methods attempted for human cardiac tissue. (**a**) Chemical-based decellularization methods tested for human cardiac tissue. The details regarding the solutions used and exposure duration applied are highlighted for each decellularization step. For each donor is shown: (**b**), (**e**), (**h**) Gross tissue morphology. Scale bars: 1 mm; (**c**), (**f**), (**i**) Hematoxylin & Eosin staining. Scale bars: 100 µm; (**d**), (**g**), (**j**) Masson’s Trichome staining. Scale bars: 100 µm.

In this study, we tested four methods to develop an appropriate protocol for human cardiac tissue decellularization (Fig. 1a) and evaluated their efficiency in three unrelated donors (1, 2 and 3; Table 1). Histological staining using H&E and Masson’s Trichrome allowed preliminary assessment of the impact of all decellularization strategies tested concerning loss of cellularity as well as ECM structure and composition preservation (Fig. 1b–j).

Even though most decellularization methods for cardiac tissue are optimized either in small or large animals, we are aware of a protocol that was tested in human myocardial tissue samples. Method A was already reported for decellularization of micrometric sheets (~ 300 µm) of human myocardium biopsies and used a low percentage of SDS (0.5%) for 6 h followed by a washing step with FBS^14^ (Fig. 1a). This method resulted in reduced removal of nuclei and overall maintenance of ECM proteins for the three donors, with limited disruption of tissue architecture (Fig. 1b–j, 2nd column). For human myocardium micrometric slices method A can yield an almost complete reduction in dsDNA content^14^, however it became evident it was not efficient for attempting decellularization of tissue pieces with about 1 mm in thickness.

**Figure 2:**
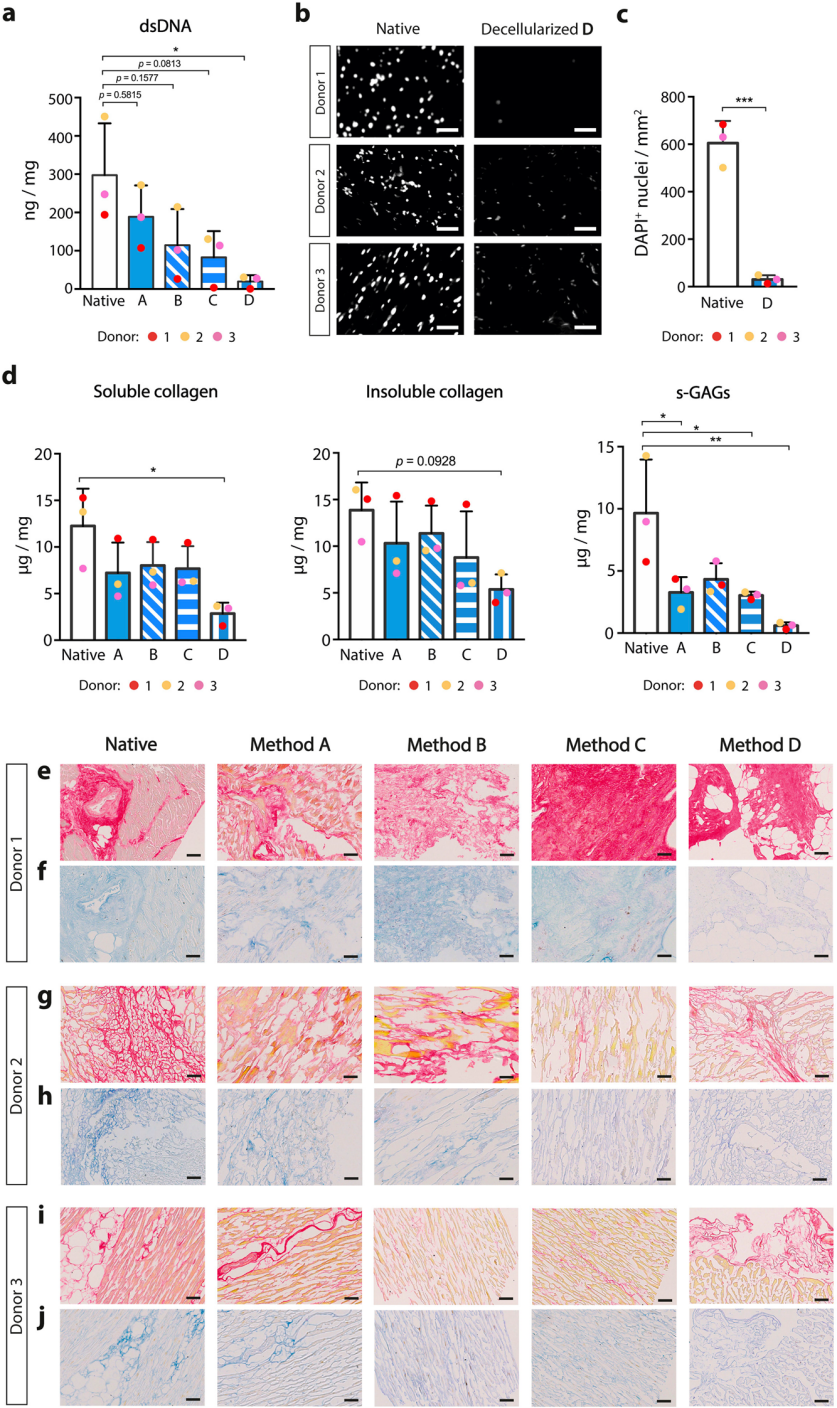
Biological characterization of chemical-based decellularized human cardiac tissue. (**a**) Double stranded DNA (dsDNA) content of native and decellularized cardiac tissue for all methods, normalized to tissue wet weight (*n* = 3; one-way ANOVA, *F*_4,10_ = 4.38, *p < 0.05 with Tukey’s multiple comparison test). (**b**) Immunofluorescence of nuclei (DAPI, white) in native and decellularized tissue through method D for each donor. Scale bars: 50 µm. (**c**) Number of DAPI + nuclei per cross-section area (*n* = 3; two-tailed unpaired *t*-test, ***p < 0.001). (**d**) Biochemical analysis for soluble collagen (*n* = 3; Kruskal–Wallis test, *p = 0.0229, *p < 0.05 with Dunnett’s multiple-comparison test), insoluble collagen (*n* = 3; one-way ANOVA, *F*_4,10_ = 2.304, following Tukey’s multiple comparison test), sulfated glycosaminoglycans (s-GAGs; *n* = 3; one-way ANOVA, *F*_4,10_ = 7.709, *p < 0.05 and **p < 0.01 with Tukey’s multiple comparison test) on native and decellularized cardiac tissue for all methods, normalized to tissue wet weight. The average of the 3 cardiac tissue donors was used as a representative value for the component in the myocardium. (**e**), (**g**), (**i**) Picro Sirius Red (PSR) and (**f**), (**h**), (**j**) Alcian Blue staining on native and decellularized tissue for all methods. Scale bars: 100 µm. Data are represented as mean ± SD.

We next tested the applicability of protocols developed for porcine cardiac tissue decellularization on human samples, which typically work well for millimeter thick tissue pieces but require a more extended exposure and higher concentration of chemical agents to achieve complete decellularization^10,11^ (i.e., Methods B and C, Fig. 1a). Method B used 1% SDS for 48 h and 1% Triton-X-100 for 30 min, and despite 2–3 days of exposure to decellularization agents being enough to remove cellular material from porcine cardiac tissue, the same did not hold for human tissue samples. In fact, donor-to-donor variability was observed when using method B; while for donor 1 there was an almost complete removal of cells and preservation of interstitial collagen matrix, for donors 2 and 3 method B was inefficient to remove the majority of cells, even though ECM architecture appeared more disorganized in comparison with method A (Fig. 1b–j, 3rd column). Incorporating an initial step to promote cell lysis can facilitate subsequent cellular membrane solubilization by detergents, as previously described for a multitude of other tissues^19^. Thus, in method C cardiac tissue pieces were firstly treated with a lysis buffer solution for 24 h, and the remainder steps of the protocol were done similarly to method B (Fig. 1a). Method C yielded inconsistent decellularization outcomes among donors; no meaningful differences were observed for donor 1 and 3 when comparing method B and C, even though donor 2 showed a reduction in nuclei staining (Fig. 1b–j, 4th column).

**Figure 3:**
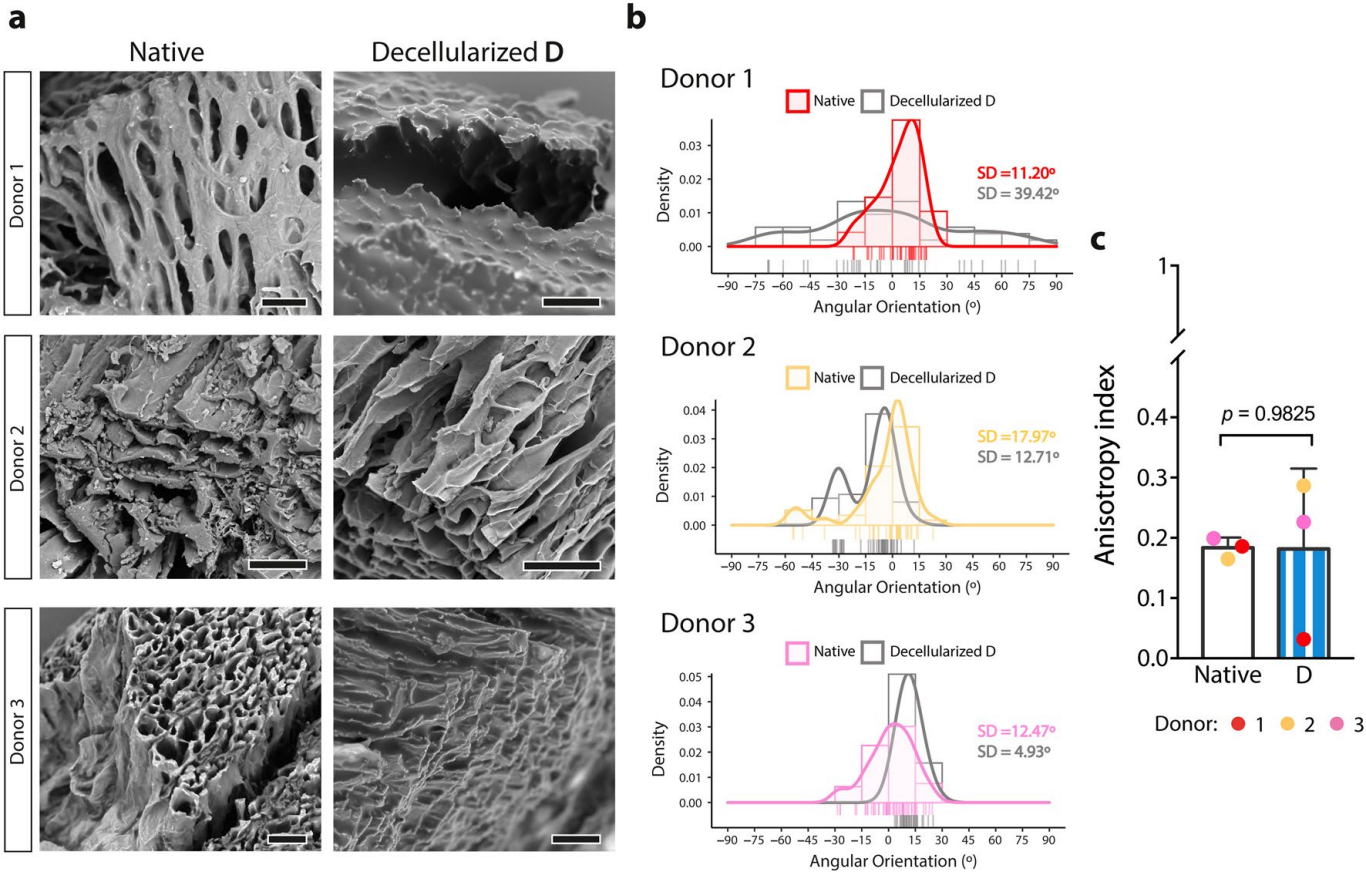
Scanning electron microscopy analysis of native and decellularized human cardiac tissue. (**a**) Architecture of native cardiac tissue and the decellularized scaffold using method D. Scale bars: 50 µm. (**b**) Density histogram of extracellular matrix fiber orientation of native and decellularized tissue using method D (*n* = 35–55 ROI analyzed per condition per tissue donor). (**c**) Anisotropy index (0—random alignment/isotropic array, 1—perfect alignment/purely anisotropic array) of native and decellularized tissue using method D (*n* = 3; two-tailed unpaired *t*-test). Data are represented as mean ± SD.

**Figure 4:**
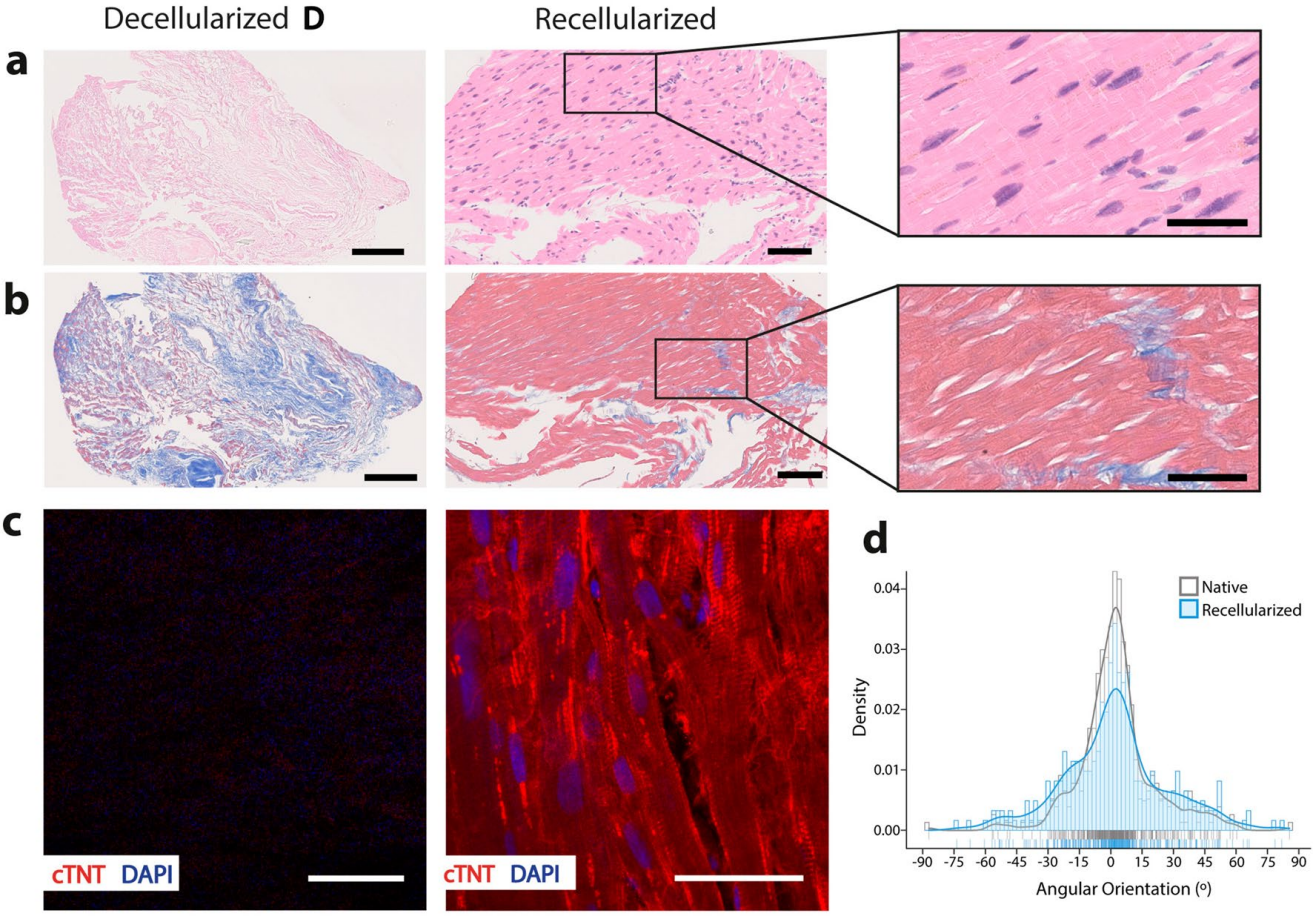
Assessment of tissue formation capacity of human induced pluripotent stem cell-derived cardiomyocytes (hiPSC-CM) on decellularized human cardiac tissue. (**a**) Hematoxylin & Eosin and (**b**) Masson’s Trichome staining of decellularized (method D) and recellularized tissue after 14 days of culture, at low (scale bars: 100 µm) and high magnification (scale bars: 50 µm). (**c**) Immunofluorescence micrograph of cardiac troponin T (cTNT, red) expression in decellularized tissue and recellularized tissue after 14 days of culture, and counterstained with DAPI (blue). Scale bar: 50 µm. (**d**) Density histogram of mean angular orientation of ventricular cardiomyocytes in native cardiac tissue and hiPSC-CM in recellularized tissue (*n* > 300 ROI analyzed per condition).

Overall, a lysis step was not sufficient to achieve a similar decellularization outcome among cardiac tissue donors, and so we sought to instead extend the exposure to detergents. Method D was performed using the same concentrations of detergents as in method B, but tissue pieces were solubilized in SDS for 192 h (8 days) followed by 24 h in Triton-X-100^8^ (Fig. 1a). For donor 1, method D completely removed cellular material and yielded a decellularized scaffold with apparent preservation of ECM fibrous components. Comparatively to methods B and C, method D seems to have further reduced nuclei staining in donors 2 and 3. Even though there is a more noticeable staining for matrisome proteins, namely collagens, in decellularized tissue sections through method D for donors 2 and 3, partially unremoved cellular components are still showing (Fig. 1b–j, 5th column).

### Characterization of decellularized extracellular matrix composition and architecture

To quantitatively assess the degree of decellularization, reminiscent double stranded DNA (dsDNA) was quantified. Native myocardial tissue contained 297.4 ± 135.5 ng/mg wet tissue of dsDNA, and only longer detergent treatment allowed a significant removal of almost all nucleic material; decellularized tissue through method D had 19.6 ± 16.7 ng/mg wet tissue of dsDNA, corresponding to a 93.4% reduction (Fig. 2a). Methods A, B and C yielded decellularization efficacies of 36.5% (188.8 ± 82.0 ng/mg wet tissue), 59.6% (114.4 ± 94.5 ng/mg wet tissue) and 72.2% (82.7 ± 68.8 ng/mg wet tissue), respectively, and dsDNA content was not considered statistically significant from native tissue. Tissue sections decellularized through method D were stained with DAPI and revealed a significant reduction of nucleic material (Fig. 2b, c).

Biochemical analysis of decellularized tissue indicated moderate decreases of soluble and insoluble collagen, and low retention of s-GAGs (Fig. 2d), especially for method D. Picro Sirius Red staining for collagen (Fig. 2e, g, i) and Alcian Blue staining for GAGs (Fig. 2f, h, j) confirmed consistent reduction of these ECM components in all tissue donors for all strategies tested, even though they are still present in decellularized tissue sections obtained using method D.

Although method D can practically remove all nucleic material and moderately retains ECM components, tissues became softer after decellularization (Fig. 1b, e, h), which could indicate partial destruction of their microarchitecture. In fact, the 3D architecture of decellularized cardiac tissue was not preserved similarly among donors after decellularization using method D (Fig. 3). Typical anisotropic interconnected lacunae were absent in donor 1, but for the remaining tissue donors structural compliance did not seem to be affected (Fig. 3a). We examined the gross alignment of ECM fibers on native cardiac tissue and compared them to the decellularized cardiac tissue through method D. On native tissue there is a low dispersion of ECM fiber orientation, with the majority occupying two to three 15° bins in density histograms (Fig. 3b). However, the same tendency is only maintained for donors 2 and 3 after decellularization. The same trend is evident with the anisotropy index of the scaffold, which is substantially decreased for donor 1 (Fig. 3c), even though method D did not statistically impact tissue anisotropy.

### Recellularization of decellularized cardiac tissue with hiPSC-cardiomyocytes

We then tested whether decellularized tissue through method D could support cell engraftment and tissue formation using cardiomyocytes derived from human induced pluripotent stem cells (hiPSC-CM), that were generated via temporal modulation of the Wnt/β-catenin pathway, as we previously described^17,18^. hiPSC-CM were seeded as single cells on decellularized tissue strips (~ 1 mm length, ~ 1 mm height) and cultured for an additional 2 weeks. These tissue strips supported hiPSC-CM attachment, ensuring efficient cell retention (Fig. 4a, b) and sustaining spontaneous contraction up to 14 days (Supplementary Video 1). Noteworthy, recellularized cardiac tissues showed positive staining for cardiac troponin T (Fig. 4c), confirming their cardiac phenotype. Furthermore, the decellularized scaffold encouraged hiPSC-CM alignment within 14 days of in vitro culture, with a mean cell angular orientation closely approaching the values measured for ventricular cardiomyocytes in native cardiac muscle (Fig. 4d).

## Discussion

Cardiac muscle is a structurally complex tissue optimized for life-long pumping function, and so it is challenging to replicate in vitro^1^. The ECM is nature’s template of a biomaterial and, through decellularization, such a scaffold could be produced to reliably engineer bioartificial cardiac tissue. Here, we aimed at evaluating several methods to efficiently decellularize human cardiac tissue, and test if the tissue scaffold could be used for tissue engineering.

Human cardiac tissue can be harvested during interventional biopsies or through segmental resection of *postmortem* hearts unsuitable for transplantation purposes. However, the vascular network of these tissue samples is often inaccessible, prohibiting perfusion of decellularization agents. In these cases, decellularization is often accomplished via tissue immersion in decellularization agents under gentle agitation to promote efficient diffusion. Among the factors that need to be considered when using this approach is tissue thickness. This provides reasoning why Method A, that was implemented on micrometer thick myocardium slices^14^, was ineffective to decellularize ~ 1 mm thick tissue pieces, that understandably have a higher cell density. An almost complete removal of nucleic material for all human cardiac tissues tested was only achieved in method D, in which the exposure times to detergents is practically equal to those used in whole-human heart perfusion-decellularization^8^. Lack of homogeneous exposure to decellularization solutions in methods using immersion is a clear limitation compared to perfusion-decellularization, and consequently prolonged exposure times are mandatory to eliminate dsDNA and other genetic material present in millimeter thick tissues. Even though mechanical damage to the ECM associated with pressure flow is minimal in immersion methods compared to perfusion-decellularization^20^, the need to extend detergent action in these methods undeniably influenced ECM biochemical composition. Quantitative analyses of some of the most prevalent ECM components, such as collagens and s-GAGs, revealed extended exposure to detergents significantly affected soluble collagen and s-GAG presence in decellularized tissue through method D. Others have also found out the retention of soluble components, such as s-GAGs^21^, soluble collagen^8^, elastin^8^ and growth factors^22^, can be severely affected using SDS/Triton-X-100 combinations, especially with extended exposure times. Conversely, insoluble collagen, that is more mature and mechanically resilient owed to cross-linking, is better retained.

Interestingly, we observed high variability when assessing the efficiency of methods B and C. Method B was sufficient to substantially reduce tissue cellularity and dsDNA content in donor 1, but the same was not verified for donors 2 and 3. Method C was an attempt to improve method B without extending detergent exposure, but was insufficient to significantly reduce cellular content in donors 2 and 3. The 48 h SDS period was based on successful protocols optimized to decellularize porcine cardiac tissue^10^. Inter-species differences may therefore prevent direct translation of protocols implemented in animals to human tissue. The reasons for such disparities may be manifold, in which tissue age may play a part. Human tissue samples are typically obtained from older donors, like the ones used here, while animal tissue can be harvested at earlier stages of life. Ageing induces structural changes in the myocardium, such as cardiomyocyte hypertrophic remodeling, increased collagen deposition and cross-linking, and increased proteolytic degradation via matrix metalloproteinase activity^23^, that could well impact the efficiency of decellularization agents. Even among animals, it has been shown SIS-ECM harvested from pigs that differ only in age presented dissimilar mechanical, structural and biological properties^24^. In addition, porcine and human cardiac ECM can simply have a distinct biological composition, and so be differentially susceptible to the type, concentration and exposure time of decellularization agents. For example, the higher lipidic content of decellularized human cardiac ECM when compared with its porcine analogue compromised in vitro gelation after solubilization^25^.

We could have further evaluated a range of ionic detergent treatment between 2 and 8/9 days, aiming at identifying an optimal protocol less harsh than method D; however, such an alternative may not exist since dsDNA was still present (in low amounts) in donors 2 and 3 after method D. Instead, combining detergents with an endonuclease may be preferable to reduce detergent exposure, even though nucleases can persist in tissue after the rinsing step^19^. Despite prolonged SDS/Triton-X-100 exposure being useful to reduced dsDNA to acceptable levels, we were unable to completely eliminate cellular components in donors 2 and 3 after method D (and to a lesser extent in donor 4; view Fig. 4b). The denaturant properties of these detergents seem to be efficient in solubilizing cell membranes, and consequently wash away nucleic material, but SDS action, in particular, was insufficient to effectively disrupt intracellular protein–protein interactions and aid their solubilization, regardless of cardiac tissue incubation with a lysis buffer solution beforehand (i.e., method C). In *postmortem* muscle tissue, as ATP is depleted, myosin heads remain bound to actin, indefinitely creating a rigor actomyosin complex that causes muscle tension and is challenging to properly solubilize^26^. This could explain why cellular remnants persisted in donors 2 and 3 after method D, which are possibly derived from cardiomyocytes’ contractile apparatus proteins and not from non-myocytes. In fact, attempting to decellularize mice left ventricle tissue with SDS alone created protein aggregates, that included macroproteins such as titin, and these were difficult to solubilize without enzymatic digestion^27^. Replacing SDS/Triton-X-100 with a trypsin/Triton-X-100 combination showed to better eliminate cellular remnants during perfusion-decellularization of thick porcine cardiac tissue slabs^28^. Nevertheless, others have reported trypsin has nonspecific degradation effects on heart ECM, particularly affecting preservation of collagen IV, laminin and elastin^29^. To overcome these issues, it has been proposed sarcolemma permeabilization followed by incubation with non-enzymatic solutions that allow sarcomere relaxation and disassembly can effectively fully decellularize rat myocardium patches, outperforming SDS-based methods^30^. Still, it remains elusive if the same protocol can be consistently applied to human tissue or whether it requires tuning.

Besides failure in using protocols established in animal tissue, human donor-to-donor variability is still apparent in our study. For donor 1, method B or C are suitable decellularization methods, showing successful removal of nucleic material and preservation of collagen content. However, when attempting to come up with a “one-size fits all” protocol (i.e., method D), the anisotropy of donor 1 was irreversibly lost alongside with noteworthy changes in its composition. For donors 2 and 3 tissue architecture was maintained and dsDNA significantly removed, yet tissue biochemical composition is thoroughly affected. Therefore, obtaining an acellular scaffold from human cardiac tissue that is both sufficiently complex and presents minimal variability might require optimization of current methods in a personalized way. The interindividual heterogeneity across human cardiac tissue is surprising but not unexpected, as a previous study already confirmed, through a global proteomics analysis, human myocardial ECM preparations show significant donor-to-donor variability^31^. The reasons behind this are challenging to discern and may be a combination of multiple factors like age, gender, and differential tissue cellular and molecular profiles, among others that warrant further investigation. Despite the low number of biological samples analyzed, our study reinforces the idea tissue-to-tissue variability should be considered when evaluating a decellularization protocol, which is often overlooked in the field. Furthermore, a compromise between cell removal and ECM preservation must be established, since for most strategies complete elimination of cells is incompatible with full retention of ECM proteins^29^, and an acellular scaffold may not even be essential if the ECM is not intended for in vivo implantation.

We further confirmed the suitability of the decellularized scaffold for human myocardium bioengineering. Previous attempts at recellularizing cardiac ECM have either used animal-derived neonatal proliferative cardiomyocytes^6,7^, non-cardiac cells^14,32^ or human uncommitted cardiac progenitor cells^33^. Fewer studies have used hiPSC-CM, and those that have attempted to recellularize human cardiac ECM with these cells have done it with low cell densities^8,14,34^, impairing efficient cell retention and robust tissue formation. In this work, we were able to fully repopulate decellularized cardiac tissue with hiPSC-CM, and the scaffold ensured cell retention, alignment and sustained spontaneous contractions up to 2 weeks in culture. In two-dimensional planar culture conditions hiPSC-CM lack proper organization and are unable to replicate the bundle-like appearance of cardiac muscle^1^. Immature neonatal cardiomyocytes are responsive to substrate stiffness, that influences twitch force and myofibril structure^35^. Substrate stiffness equally tunes the mechanical output of hiPSC-CM, which is improved when it matches the physiological stiffness of the myocardium^36^. Although further studies are needed, our decellularized human cardiac scaffold may be exerting a similar effect, thus improving the mechano-microenvironment of hiPSC-CM, and possibly features of structural maturation^16^.

Taken all together, producing an ECM preparation from human cardiac tissue requires a protocol tailored to the donor, with specific criteria outlining how biomimetically faithful the scaffold needs to be as to find a balance between acellularity and ECM compositional and architectural preservation. This perspective is in line with the goals of personalized medicine, ensuring product quality when designing an ECM biomaterial that is adequate for each experimental or clinical scenario. For tissue bioengineering applications we envision method D suffices; however, to study the cardiac ECM’s role during differentiation^4^ or how an aged^37^ or diseased^38^ ECM contributes to alteration in cardiac phenotype, the harshness of method D might compromise product quality attributes and other decellularization strategies should be pursued. Despite how tempting the notion of protocol standardization is, for human tissue it is likely unfeasible.

## Supplementary Information

Supplementary Legends.

Supplementary Video S1.
