# Supplementary material for: Interindividual heterogeneity affects the outcome of human cardiac tissue decellularization
Source: Sci Rep. 2021 Oct 21;11:20834. doi: 10.1038/s41598-021-00226-5 (PMC8531368; doi:10.1038/s41598-021-00226-5)
Supplement: Supplementary file 1 — Supplementary Legends. [file 41598_2021_226_MOESM1_ESM.docx]

**Supplementary material**

**Interindividual heterogeneity affects the outcome of human cardiac tissue decellularization**

Miguel F. Tenreiro^1,2^, Henrique V. Almeida^1,2^, Tomás Calmeiro^3^, Elvira Fortunato^3^, Lino Ferreira^4,5^, Paula M. Alves^1,2^, Margarida Serra^1,2^*

^1^iBET, Instituto de Biologia Experimental e Tecnológica, Apartado 12, 2781-901 Oeiras, Portugal;

^2^Instituto de Tecnologia Química e Biológica António Xavier, Universidade Nova de Lisboa, Av. da República, 2780-157 Oeiras, Portugal;

^3^CENIMAT|i3N, Departamento de Ciência dos Materiais, Faculdade de Ciências e Tecnologia, Universidade NOVA de Lisboa, Campus de Caparica, 2829-516 Caparica, Portugal.

^4^CNC, Centro de Neurociências e Biologia Celular, Universidade de Coimbra, 3004-517 Coimbra, Portugal;

^5^Faculdade de Medicina, Universidade de Coimbra, Rua Larga, 3004-504 Coimbra, Portugal.

*- corresponding author: Margarida Serra, iBET, Instituto de Biologia Experimental e Tecnológica, Apartado 12, 2780-901 Oeiras, Portugal, Telephone: +351 21 446 94 31; e-mail: [mserra@ibet.pt](mailto:mserra@ibet.pt);

**Supplementary Video 1 |** Spontaneously beating hiPSC-CM on decellularized human cardiac tissue after 14 days of culture.
